# Identifying with the beautiful: Facial attractiveness effects on unisensory and multisensory self–other distinction

**DOI:** 10.1177/17470218211050318

**Published:** 2021-10-13

**Authors:** Elena Panagiotopoulou, Laura Crucianelli, Alessandra Lemma, Aikaterini Fotopoulou

**Affiliations:** 1 Research Department of Clinical, Educational and Health Psychology, University College London, London, UK; 2 Postgraduate Studies, Anna Freud National Centre for Children and Families, London, UK; 3 Department of Neuroscience, Karolinska Institutet, Stockholm, Sweden

**Keywords:** Facial attractiveness, multisensory integration, enfacement illusion, self-enhancement bias

## Abstract

People tend to evaluate their own traits and abilities favourably and such favourable self-perceptions extend to attractiveness. However, the exact mechanism underlying this self-enhancement bias remains unclear. One possibility could be the identification with attractive others through blurring of self–other boundaries. Across two experiments, we used the enfacement illusion to investigate the effect of others’ attractiveness in the multisensory perception of the self. In Experiment 1 (*N* = 35), participants received synchronous or asynchronous interpersonal visuo-tactile stimulation with an attractive and non-attractive face. In Experiment 2 (*N* = 35), two new faces were used and spatial incongruency was introduced as a control condition. The results showed that increased ratings of attractiveness of an unfamiliar face lead to blurring of self–other boundaries, allowing the identification of our psychological self with another’s physical self and specifically their face, and this seems to be unrelated to perceived own attractiveness. The effect of facial attractiveness on face ownership showed dissociable mechanisms, with multisensory integration modulating the effect on similarity but not identification, an effect that may be purely based on vision. Overall, our findings suggest that others’ attractiveness may lead to positive distortions of the self. This research provides a psychophysical starting point for studying the impact of others’ attractiveness on self-face recognition, which can be particularly important for individuals with malleable, embodied self–other boundaries and body image disturbances.

## Introduction

People tend to perceive themselves in a positive manner, displaying attributional, memorial, and evaluative biases that favour the self (Sedikides & Green, 2000). For instance, a pervasive bias in learning, thought to be arising from self-enhancing motivations, leads people to change their beliefs about the future more readily when confronted with good news rather than bad news (Sharot & Garrett, 2016). They also tend to evaluate their own traits and abilities favourably, creating flattering images of themselves (Dunning, 1999) and such favourable self-perceptions extend to physical attractiveness. A high level of agreement across individuals, cultures, and age groups has been found in terms of how attractiveness is perceived, yet there is still a debate about whether this reflects an innate preference (Langlois et al., 1987), or common learning as a result of adaptation (e.g., Hahn & Perrett, 2014). Crucially, facial attractiveness appears to be highly valued in social exchanges, with evidence pointing to an advantage for attractive people in terms of how others perceive and judge them in social interactions and beyond (Langlois et al., 2000; Mobius & Rosenblat, 2006; Riniolo, Johnson, Sherman, & Misso, 2006; Tartaglia & Rollero, 2015; Zebrowitz, Hall, Murphy, & Rhodes, 2002). Research has shown that people consider themselves more attractive than the average person (Horton, 2003). They also make self-enhancing judgements for their own attractiveness even when they rate themselves to be more overweight than others consider them to be, indicating a dissociation between self-perception of body image and physical attractiveness (Donaghue & Smith, 2008). This self-serving bias seems to also extend to face recognition, an ability which is considered an index of self-awareness and a fundamental aspect of the sense of selfhood (Gallup, 1970; Rochat, 2009). In a study by Epley and Whitchurch (2008), participants’ faces were made more or less attractive using a morphing procedure, and participants were found to be more likely to recognise an attractively enhanced version of their face as their own. The results suggest that the recognition of one’s own face as being more attractive than it actually is, represents a distinct form of self-enhancement, produced by relatively implicit and automatic psychological mechanisms. Moreover, this self-enhancement bias was correlated with implicit measures of self-worth and it was, therefore, suggested that this may reflect a top–down effect of making positive associations to the self, ultimately leading to positive distortions of it. However, the exact mechanism underlying this self-enhancement bias for face recognition remains unclear. Horton (2003) did not only show that participants regarded themselves as more attractive than the average person, but also more similar to attractive targets. Therefore, a plausible mechanism underlying this self-enhancement bias for face recognition could be a blurring of bodily boundaries between self and attractive others, resulting in the identification with attractive faces.

Self–other merging has been found to occur as a result of synchronous multisensory stimulation. Multisensory integration, defined as the ability of the brain to synthesise information across modalities, is fundamental for self-perception and the bodily self, more generally (Blanke, 2012; Ehrsson, 2012). Multisensory integration paradigms allow the manipulation of the perception of one’s own limb (Rubber Hand Illusion, Botvinick & Cohen, 1998) or body (“full body illusion,” (Ehrsson, 2007; Lenggenhager, Tadi, Metzinger, & Blanke, 2007), by blurring self–other boundaries. In such bodily illusions, temporal and spatial congruency between seen and felt sensory events gives rise to the sense of body ownership, that is, the feeling that a body (part) belongs to me (Tsakiris & Haggard, 2005). The same is also true for the face, which is probably the most representative instance of personal identity (Filippetti, Orioli, Johnson, & Farroni, 2015). Synchronous multisensory stimulation between two faces gives rise to the “enfacement illusion,” with participants assimilating more of the other person’s features in their own self-face representation (Paladino, Mazzurega, Pavani, & Schubert, 2010; Sforza, Bufalari, Haggard, & Aglioti, 2010; Tsakiris, 2008). The “enfacement illusion” extends beyond body perception to a more conceptual merging between self and other, by affecting social cognition processes (Paladino et al., 2010) and affective ratings for the other face, including ratings of greater attractiveness and trustworthiness after the induction of the illusion (Tajadura-Jiménez, Longo, Coleman, & Tsakiris, 2012). As such, the enfacement illusion paradigm affords us the opportunity to experimentally manipulate the effects of attractiveness in a simulated situation of social interaction. The strength of the illusion has been found to positively correlate with physical attractiveness attributed to the partner’s face (Sforza et al., 2010). However, this study could only establish a correlation between facial attractiveness and enfacement given that facial attractiveness was not experimentally manipulated using both attractive and unattractive faces but rather merely measured on the basis of subjective ratings and then correlated with enfacement scores. The partners of that study were also familiar to each other, hence it cannot be ruled out that perceived attractiveness may have been influenced by other factors, such as familiarity and social desirability. The question, therefore, remains as to whether other people’s attractiveness may influence self-face recognition during multisensory integration and whether the underlying mechanism is a top–down process (e.g., such as beliefs and desirability associated with attractiveness) or a bottom–up multisensory integration effect of attractiveness (e.g., bottom–up aspects of stimuli, such as fluency, symmetry and salience). Although the distinction between bottom–up and top–down processes in the brain’s hierarchy is a complex and debated issue, in this particular case, we employ the terms to refer specifically to information processing that is guided by an individual’s higher-level knowledge and related conscious or unconscious expectations (top–down processes) and processes that take sensory information from the environment and transform it into neural impulses without recourse to an individual’s prior knowledge (bottom–up processes). The latter may, for example, involve bottom–up aspects of attention that can be driven by inherent properties of a stimulus relative to its background, rather than the individual’s goals or expectations. For instance, attractive faces have been found to capture greater spatial and temporal attention (Liu & Chen, 2012; Nakamura & Kawabata, 2014; Sui & Liu, 2009) and at least some of these attentional effects do not seem to relate to individuals’ expectations and goals about beauty but seem rather rapid and automatic (Palermo & Rhodes, 2007; Sui & Liu, 2009). This idea is partially supported by a previous study showing that attractiveness seems to play a crucial role in the enhancement of tactile perception (i.e., visual remapping of touch [VRT]; Serino, Pizzoferrato, & Làdavas, 2008) on the face when observing a more or less attractive avatar (Noel, Giovagnoli, Costa, & Serino, 2014). In other words, we seem to be able to transfer the attribution of physical attractiveness to our own multisensory perception system to some extent.

To this end, the literature points to a self-enhancement bias, by which people perceive their own face as more attractive than it actually is. However, the exact mechanism underlying this remains unclear and a possibility could be the identification with others’ attractive faces through blurring of self–other boundaries. Accordingly, here we aim to elucidate whether being exposed to other people’s faces, either attractive or unattractive, can modulate the way we perceive ourselves. To address this question, this study used the enfacement illusion paradigm to examine for the first time the role of attractiveness in the multisensory modulation of face ownership over two experiments. Although the term “self-face recognition” is habitually used as the face analogue of “body ownership,” previous research has shown a dissociation between explicit self-report measures (enfacement questionnaire) and implicit measures (self-recognition task; Panagiotopoulou, Filippetti, Tsakiris, & Fotopoulou, 2017). Therefore, the term “face ownership” is used instead to describe both explicit and implicit aspects of enfacement, with identification and similarity referring to the explicit self-report component and self-face recognition referring to the implicit component. In a first experiment (*N* = 35), participants were stroked on the cheek while they were seeing an attractive or a non-attractive face being stroked on the cheek in synchrony or asynchrony. Participants were asked to complete a self-face recognition task (implicit) before and after the induction of the illusion, as well as an enfacement questionnaire (explicit self-report), after the induction of the illusion. In the second experiment (*N* = 35), two new faces were used and spatial incοngruency (cheek vs. forehead) was introduced as an alternative control condition instead of temporal asynchrony. Higher levels of enfacement were expected for an attractive vs. a non-attractive face, particularly in the synchronous (Experiment 1) and the spatial congruent (Experiment 2) condition, suggesting that attractiveness has an effect on the multisensory integration process itself, rather than being a more general, top–down effect.

## Experiment 1

### Method

In these studies, we report all measures, manipulations, and exclusions.

#### Participants

Thirty-five Caucasian female participants (*M* age 24.30 ± 3.13 *SD* years) with no psychiatric or neurological history were recruited online via a University Subject Pool system and took part in a single 1-hr experimental session in a laboratory setting. The sample size was determined before any data analysis based on prior calculations for 99% power (effect size *f* set at 0.34, G*Power 3.1) in accordance with the effect size obtained in the significant interaction in Panagiotopoulou et al. (2017) (η^2^ = 0.102). Females were tested, given that the experimenter delivering the touch was female and there is evidence suggesting that the hedonic value of touch varies according to the gender of both giver and receiver (Gazzola et al., 2012). Participants were reimbursed for their time with either payment (£10) or course credits. Written, informed consent was obtained from all participants prior to their participation. The study was approved by the Ethics Committee of the Research Department of Clinical, Educational and Health Psychology, University College London.

#### Design

The study employed a 2×2 within-subjects design with two factors: (1) Synchrony (synchronous tactile stimulation vs. asynchronous tactile stimulation) and (2) Attractiveness (other attractive face vs. other non-attractive face). The dependent measures were: (1) a self-recognition task as an implicit measure of the illusion that was delivered before and after the interpersonal stimulation and (2) an enfacement questionnaire capturing the explicit self-report experience of the illusion that was delivered only after the stimulation (see “Materials” section for details on selection of faces etc.).

### Materials

#### Facial attractiveness survey

To select the attractive and non-attractive faces for the visuo-tactile stimulation videos (described below), a survey was conducted with a separate sample of 65 Caucasian women (*M* age = 28.68, *SD* = 11.64; see Supplementary Material A for more details).

#### Construction of the visuo-tactile stimulation videos

For the induction of the illusion, two visuo-tactile stimulation video clips were created. The two females, the faces of whom were selected from the above survey to represent the attractive and non-attractive face, respectively, were invited to the lab at University College London to create the two videos. Each of these videos displayed the (attractive or non-attractive) face being stroked on the cheek with a soft cosmetic brush. Each stroke covered a distance of 8 cm in 1 s. Each video lasted 120 s; 1 s of tactile stimulation followed by 1 s of rest (60 strokes in total).

#### Construction of morphing movies for the self-recognition task

**Figure 1.**
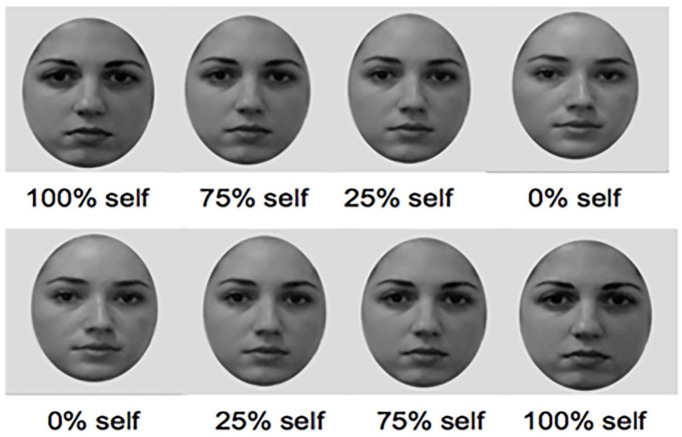
Illustrative example of self-recognition task with the selected attractive face (top right and bottom left).

For the self-recognition task, morphing movies were created for each participant. A digital photograph of the participant was taken at the beginning of the experimental session. The participant’s face in the photograph was mirror-transposed, converted to greyscale, and all non-facial attributes were removed (e.g., background, hair, ears) with GNU Image Manipulation Program (GIMP). A computerised morphing procedure implementing a mesh warping algorithm (Abrasoft Fantamorph) was used to merge each participant’s face with the unfamiliar face (attractive and less attractive) in 1% steps resulting in 100 frames with graded blending of the facial features of the two faces. For each participant, four morphing movies were created as there were two unfamiliar faces (attractive vs. non-attractive) and two directions: from 100% self to 0% self (“*self to other*” direction) and from 0% self to 100% self (“*other to self*” direction). Each movie lasted 33 s and contained 100 frames (see Figure 1).

#### Enfacement questionnaire (explicit self-report measure)

After the interpersonal stimulation, participants were asked to complete a previously used enfacement questionnaire (Panagiotopoulou et al., 2017) consisting of eight questions presented in a random order (7-point Likert-type scale; −3, *strongly disagree*; +3, *strongly agree*), which reflected participants’ explicit self-report experience of the illusion. The questionnaire used consisted of three sub-components: *identification*, that is the extent to which participants feel that the other’s face is theirs (Items 1–3, 6); *similarity*, that is the extent to which participants perceive the other’s face as similar to theirs (Items 4, 5); and *affect*, that is the extent to which participants judge the other’s face as attractive and trustworthy (Items 7, 8).

#### Procedure

**Figure 2.**
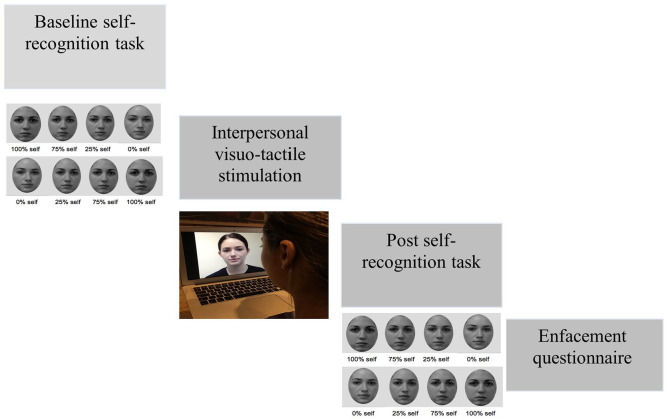
Experimental procedure per condition in Experiment 1.

Computer-generated stimulation was controlled by a customised software program (Presentation software, Neurobehavioral Systems Inc.) and presented on the screen, which was placed at a viewing distance of approximately 50 cm. The experimental session began with a baseline self-recognition task, where participants were presented with two morphing movies showing: (1) their own face morphing into one of the attractive or non-attractive faces (“self to other” direction) and (2) the attractive or non-attractive face morphing into their own face (“other to self” direction). Both videos were presented to participants in random order. Participants were asked to press the space key with their right index finger, as soon as they thought that the face shown began to look more like the face that it was morphing into (self or other depending on direction of movie). The number of seconds at which the movie was stopped was recorded. Following this baseline self-recognition task, participants were instructed to look at the screen placed in front of them, relax, and watch the visuo-tactile stimulation video for 120 s. As soon as the video began, tactile stimulation was delivered by the experimenter with a cosmetic-like soft brush on a specular congruent location between both faces either synchronously or asynchronously (with 1 s delay). Right after the task, participants completed the same self-recognition task as in baseline, as well as the enfacement questionnaire. In total, there were four conditions: (1) *attractive* face with *synchronous* stimulation; (2) *non-attractive* face with *synchronous* stimulation; (3) *attractive* face with *asynchronous* stimulation; (4) *non-attractive* face with *asynchronous* stimulation. The order of these conditions was randomised. Between conditions, participants were instructed to look at their own face for 90 s using Photobooth application for Mac computers to “break” the enfacement illusion and in preparation for the next block (see Figure 2).

#### Data analysis

All of the statistical analyses were performed using the Statistical Package for the Social Sciences (SPSS) version 23 (IBM, Chicago, IL, USA). The overall enfacement score was calculated based on the first two subcomponents (identification and similarity), and the four individual sub-components were also analysed separately, with the last two items comprising the “affect” subcomponent (i.e., attractiveness and trustworthiness) acting as manipulation checks. Separate, repeated-measures analysis of variance (ANOVA) were performed on the overall and the subcomponent scores with synchrony (synchronous vs. asynchronous) and attractiveness (attractive vs. non-attractive) as within-subject factors. Bonferroni-corrected post hoc analyses were conducted when appropriate.

For the analysis of the self-recognition task, the means of seconds at which participants stopped the videos were converted into percentage of frames containing the “self.” Given that there is evidence that the ability for self–other discrimination is influenced independent of the direction of morphing videos (Heinisch, Dinse, Tegenthoff, Juckel, & Brüne, 2011; Heinisch, Krüger, & Brüne, 2012; Payne & Tsakiris, 2017), the two directions of morphing (“self to other” and “other to self”) were averaged. As variable baseline enfacement scores have been noted in the previous research using the same task (Panagiotopoulou et al., 2017) and also repeated measures (Level 1) were nested within individuals (Level 2), multilevel modelling was implemented. First, a linear mixed model (LMM) was performed to explore the effects of attractiveness on “pre” scores, with “pre” score as the outcome variable, “attractiveness” as a dummy-coded categorical predictor, and subjects specified as random effects. Subsequently, based on the results of the above model that, as predicted, showed that baselines scores differed not only within and between individuals but also on the basis of the attractiveness manipulation, a second LMM was performed with “post” score as the outcome variable and “pre” score as the continuous predictor, mean-centred to avoid multicollinearity (Tabachnick & Fidell, 2007). “Synchrony” and “attractiveness” conditions were inserted in the models as dummy-coded categorical predictors. In all of the analyses, fixed main effects for each of the categorical and continuous explanatory variables were specified, as well as the interaction term between synchrony and attractiveness. Random intercepts for subjects were also specified (i.e., random effects).

### Results

#### Explicit self-report enfacement

##### Overall enfacement

**Figure 3.**
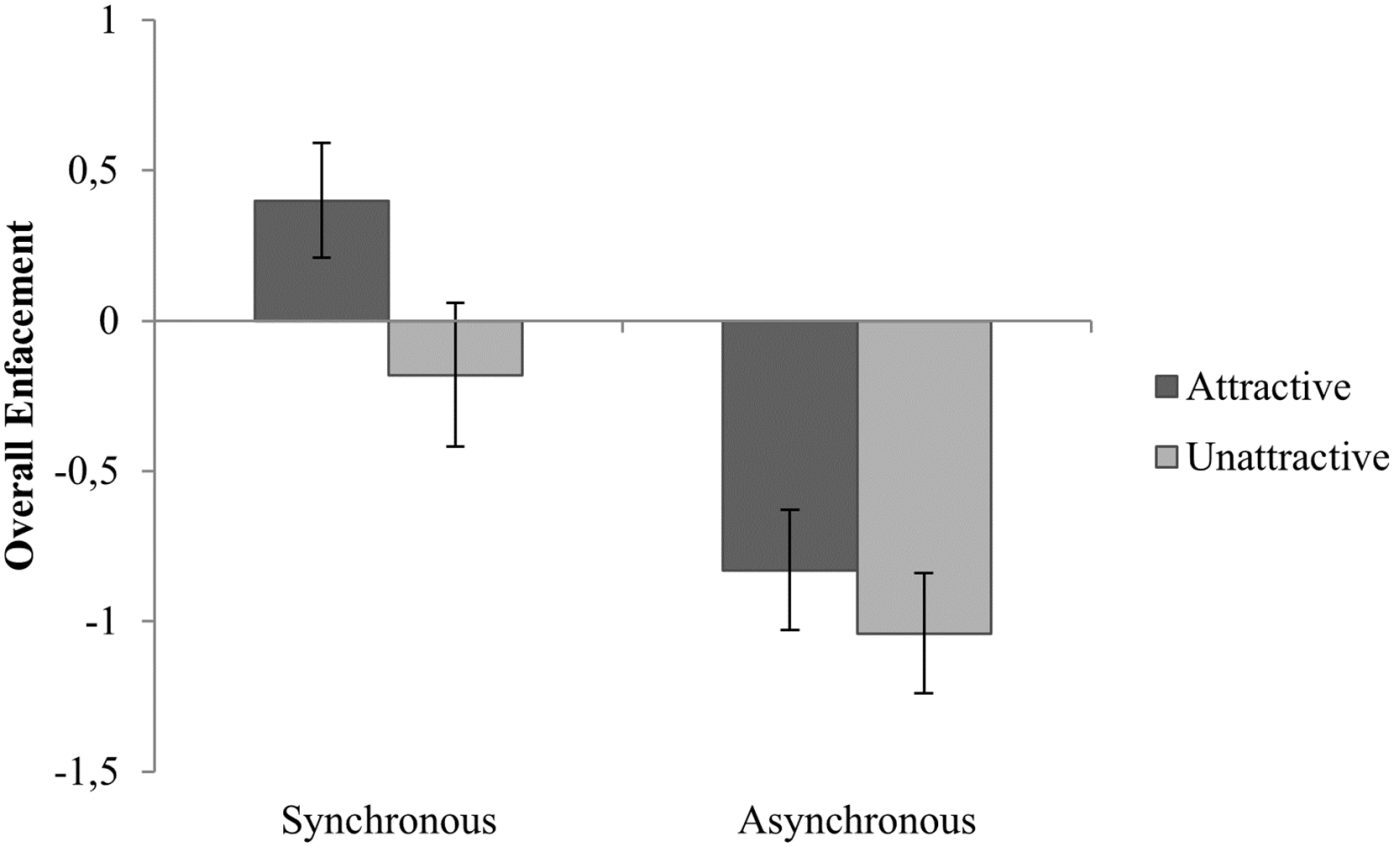
Means for overall explicit enfacement in Experiment 1. Higher scores indicate higher levels of enfacement. Error bars denote standard errors.

A 2 × 2 ANOVA revealed a significant main effect of “synchrony,” *F*(1, 34) = 47.27, *p* < .001, η^2^ = 0.582, with synchronous stroking (*M* = 0.107, *SE* = 0.190) producing higher levels of enfacement as compared with asynchronous stroking (*M* = −0.933, *SE* = 0.185). A significant main effect was also found for “attractiveness,” *F*(1, 34) = 6.48, *p* = .016, η^2^ = 0.160, with attractive face (*M* = −0.214, *SE* = 0.172) producing higher levels of enfacement as compared with non-attractive face (*M* = −0.612, *SE* = 0.204). The interaction between “synchrony” and “attractiveness” was not significant, *F*(1, 34) = 3.01, *p* = .092, η^2^ = 0.081 (Figure 3).

##### Sub-component analysis: identification

**Figure 4.**
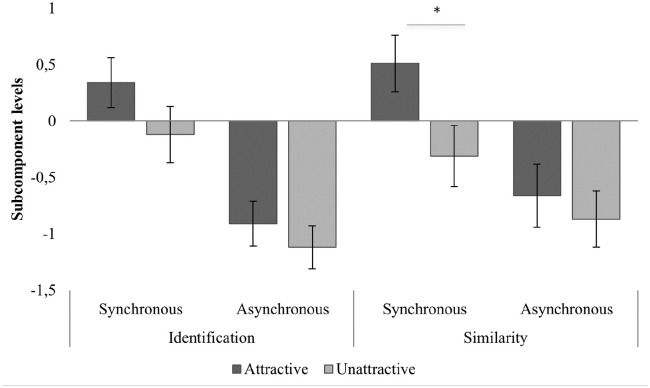
Means for identification and similarity in Experiment 1. Higher scores indicate higher levels of each sub-component. Error bars denote standard errors.

A 2 × 2 ANOVA revealed a significant main effect of “synchrony,” *F*(1, 34) = 49.07, *p* < .001, η^2^ = 0.591, with synchronous stroking (*M* = 0.111, *SE* = 0.204) producing higher levels of identification as compared with asynchronous stroking (*M* = −1.018, *SE* = 0.172). A significant main effect was also found for “attractiveness,” *F*(1, 34) = 4.52, *p* = .041, η^2^ = 0.117, with attractive face (*M* = −0.286, *SE* = 0.182) producing higher levels of identification as compared with non-attractive face (*M* = −0.621, *SE* = 0.194). The interaction between “synchrony” and “attractiveness” was not significant, *F*(1, 34) = 1.01, *p* = .322, η^2^ = 0.029 (Figure 4).

##### Sub-component analysis: similarity

A 2 × 2 ANOVA revealed a significant main effect of “synchrony,” *F*(1, 34) = 19.36, *p* < .001, η^2^ = 0.363, with synchronous stroking (*M* = 0.100, *SE* = 0.211) producing higher levels of similarity as compared with asynchronous stroking (*M* = −0.764, *SE* = 0.242). A significant main effect was also found for “attractiveness,” *F*(1, 34) = 6.07, *p* = .019, η^2^ = 0.151, with attractive face (*M* = −0.071, *SE* = 0.220) producing higher levels of similarity as compared with non-attractive face (*M* = −0.593, *SE* = 0.241). The interaction between “synchrony” and “attractiveness” was also significant, *F*(1, 34) = 4.73, *p* = .037, η^2^ = 0.122. Bonferroni-corrected post hoc tests (α = .025) revealed that perceived similarity was higher for attractive vs. unattractive face when the stimulation was synchronous, *t*(34) = 2.88, *p* = .007, *d* = 0.548, but not asynchronous, *t*(34) = 0.991, *p* = .329, *d* = 0.137 (Figure 4).

For the manipulation checks on trustworthiness and attractiveness, see Supplementary Material B.

##### Implicit self-recognition task

**Figure 5.**
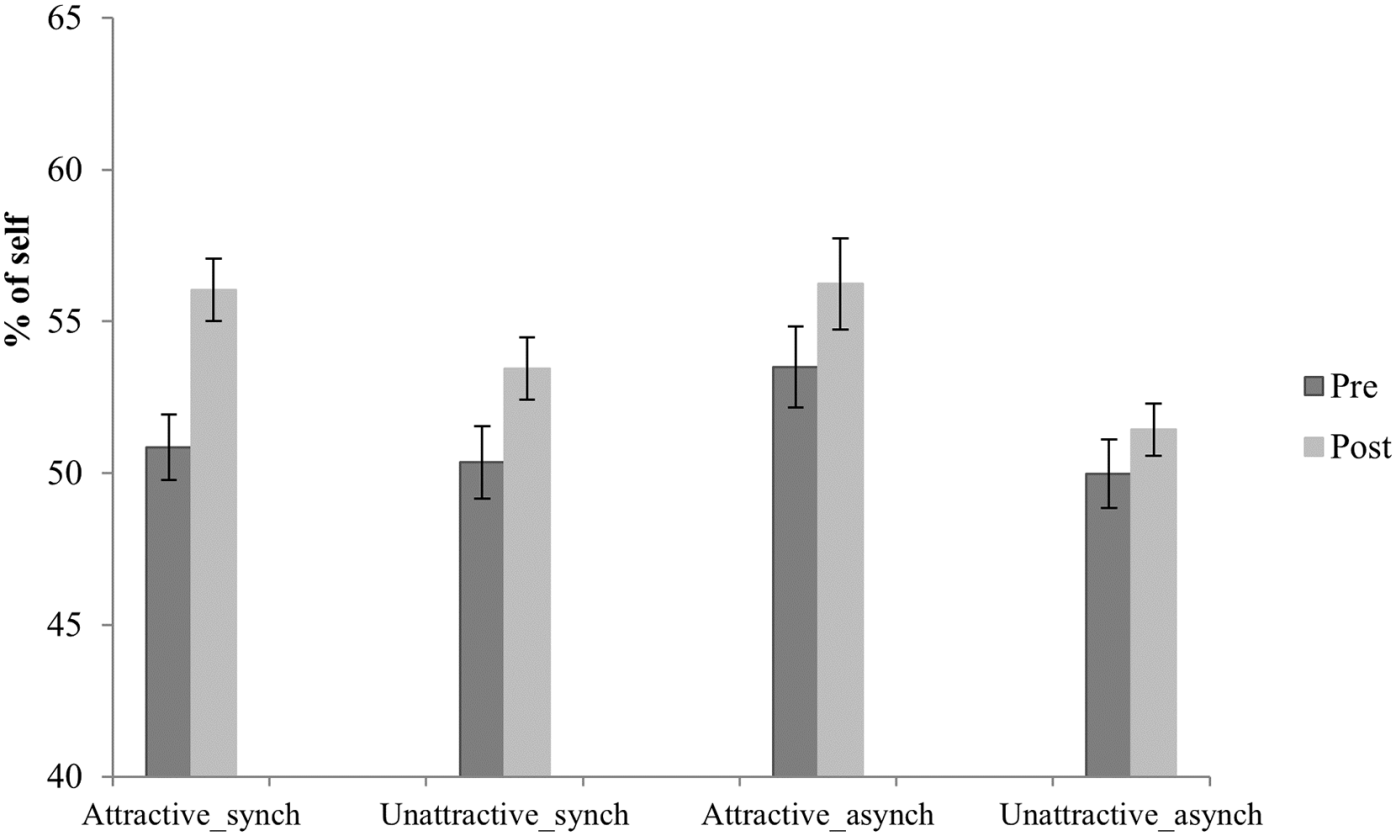
Means for percentage of frames containing “self” in the different conditions. Error bars denote standard errors.

Figure 5 illustrates the levels of implicit enfacement per condition.

The first LMM revealed a significant effect of attractiveness on “pre” score (*b* = −4.71, *SE* = 0.808, *p* < .001), with attractive face (*M* = 52.17, *SD* = 6.15) leading to higher levels of implicit enfacement, as compared with unattractive face (*M* = 50.16, *SD* = 6.81).

The results for the second LMM with “post” score as the outcome variable are presented in the table below:

As reported in Table 1, there was a significant main effect of synchrony with synchronous stimulation (*M* = 54.046, *SD* = 0.845) leading to higher levels of implicit enfacement as compared with asynchronous stimulation (*M* = 51.11, *SD* = 0.716). The main effect of attractiveness and the interaction between attractiveness and synchrony were non-significant.

**Table 1.** Multilevel modelling results for outcome variable “post” scores.

Effect	*b*	*SE*	*p*-value	95% confidence interval	
				Lower bound	Upper bound
Synchrony	**3.459247**	**1.247233**	**.009**	**0.930959**	**5.987536**
Attractiveness	0.132677	1.006735	.896	−1.913567	2.178921
Synchrony × Attractiveness	−0.206752	1.693915	.904	−3.651071	3.237566
Pre	**0.516553**	**0.074970**	**.000**	**0.367739**	**0.665367**

*SE*: standard error. Significant main effects and interactions are highlighted in bold.

### Discussion

The findings of this first experiment showed that synchronous stimulation led to higher levels of enfacement, measured both at an implicit (self-recognition task) and an explicit, self-report level (questionnaire), thus replicating the “enfacement illusion” and confirming the important role of multisensory integration in face ownership (Paladino et al., 2010; Sforza et al., 2010; Tsakiris, 2008). The main hypothesis regarding the role of facial attractiveness in face ownership was, however, only partly confirmed, in the sense that attractiveness had dissociable effects on unisensory and multisensory perception during the enfacement illusion. These findings are discussed in detail below.

In terms of implicit face ownership (self-face recognition task), no main effect of attractiveness or interaction between synchrony and attractiveness was found. Yet, attractiveness was found to enhance implicit enfacement at baseline, prior to any interpersonal visuo-tactile stimulation. In other words, participants “enfaced” more the attractive, as compared with the non-attractive face, just by looking at it, independently of any multisensory visuo-tactile process. Previous research on other bodily illusions has shown that congruent visuo-proprioceptive cues may be sufficient to induce subjective embodiment of a fake body (part), in the absence of visuo-tactile integration, a phenomenon known as “visual capture of ownership” (VOC; Carey, Crucianelli, Preston, & Fotopoulou, 2019; Crucianelli, Krahé, Jenkinson, & Fotopoulou, 2018; Martinaud, Besharati, Jenkinson, & Fotopoulou, 2017; Ponzo, Kirsch, Fotopoulou, & Jenkinson, 2018). The finding of the current experiment suggests that attractiveness may have a first effect on enfacement purely based on vision and irrespective of multisensory integration.

With regard to explicit self-report enfacement, a main effect of attractiveness was found for overall explicit enfacement, as well as the individual sub-components of identification and similarity. After interpersonal multisensory stimulation, the levels of overall enfacement, as well as identification (i.e., first sub-component) were significantly higher for an attractive face as compared with a non-attractive face, but no interaction between synchrony and attractiveness was found in line with the implicit findings. During synchronous stimulation, the ratings for overall enfacement and identification were higher for the attractive face. On the contrary, the ratings for overall enfacement and identification were negative when the stimulation was asynchronous, indicating the absence of enfacement, yet they were less negative when watching the attractive face as compared with the non-attractive. The absence of a significant interaction does not warrant any inferences regarding such differences between attractive vs. non-attractive face beyond this sample, yet we note here that we have found a similar pattern of results in a previous study where pleasant, affective touch was found to increase feelings of enfacement during synchronous stimulation and decrease feelings of non-enfacement during asynchronous stimulation (Panagiotopoulou et al., 2017). One possible explanation is that an affective, social stimulus—previously affective touch and, in this case, an attractive face—may not only increase identification during optimal conditions of synchronous sensory stimulation but may also have the potential to reduce “deafference,” which is described as a phenomenon of unpleasant and numb feelings about the body caused by the temporal mismatch between seen and felt tactile stimulation (Longo, Schuur, Kammers, Tsakiris, & Haggard, 2008). On the contrary, this study found an interaction between synchrony and attractiveness for explicit self-report similarity ratings (i.e., second sub-component), indicating that attractiveness led to higher levels of similarity in the synchronous condition rather than the asynchronous. Dissociable effects on identification and similarity have been previously reported (Panagiotopoulou et al., 2017), hence suggesting that identification and similarity are possibly mediated by different mechanisms (discussed in “General discussion” section in more detail).

More generally, previous research has found a different effect of affective touch on face ownership under temporal mismatch (orthogonal effects of affective touch and synchrony on multisensory integration) as compared with spatial mismatch (effect of affective touch dependent on spatial congruence; Panagiotopoulou et al., 2017). Synchrony and spatial congruency during multisensory stimulation lead to a perceptual binding between seen and felt events, while the effects of multimodal asynchronous stimulation may be dependent on various other mechanisms (Abdulkarim & Ehrsson, 2016; Rohde, Luca, & Ernst, 2011), one such being “deafference” (Longo et al., 2008). To this end, we decided to conduct a second experiment to explore whether, similar to affective touch, the effect of facial attractiveness on face ownership is also dependent on spatial congruency. To achieve this, spatial incongruency (cheek vs. forehead) was introduced as an alternative to temporal asynchrony to control for the phenomenon of “deafference” found predominantly during asynchronous stimulation. Thus, based on the results of our previous research (Panagiotopoulou et al., 2017), it was expected that facial attractiveness would lead to higher levels of enfacement during spatially congruent but not incongruent stimulation. Moreover, given the lack of baseline ratings for the explicit self-report measure in this first experiment, the enfacement questionnaire was administered both at baseline and post-stimulation and, in line with the findings of Experiment 1, it was hypothesised that attractiveness would enhance explicit enfacement even at baseline, prior to any interpersonal visuo-tactile stimulation.

## Experiment 2

### Method

#### Participants

Thirty-five Caucasian female participants (*M* age 20.89 ± 2.74 *SD* years) with no psychiatric or neurological history were recruited online via a University Subject Pool system and took part in a single 30-min experimental session in a laboratory setting. The sample size was determined before any data analysis based on prior calculations for 99% power (effect size *f* set at 0.37, G*Power 3.1) in accordance with the effect size obtained in the significant interaction between synchrony and attractiveness in Experiment 1 (η^2^ = 0.102). Participants were reimbursed for their time with either payment (£5) or course credits. Written informed consent was obtained from all participants prior to their participation. The study was approved by the Ethics Committee of the Research Department of Clinical, Educational and Health Psychology, University College London.

#### Design, materials, and procedure

**Figure 6.**
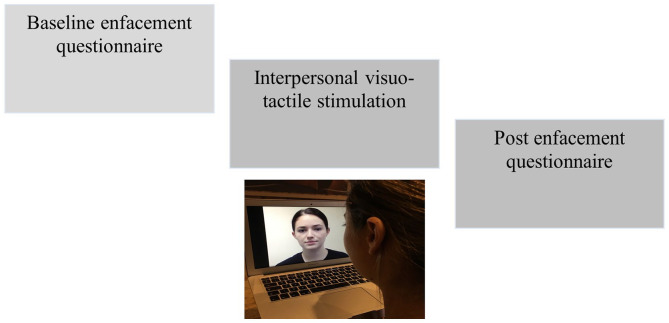
Experimental procedure per block in Experiment 2.

Design, materials, and procedures were identical to Study 1, except for the following four differences:

1. To ensure that the results of the first experiment were not down to some characteristic of the selected faces, two new faces were used in the visuo-tactile stimulation videos, once again selected on the basis of the results of an independent survey with a separate sample of 25 Caucasian women (*M* age = 25.42, *SD* = 9.43). For more details, see Supplementary Material C.
2. Spatial incongruence was used as a control instead of asynchrony. In half of the trials, participants were touched on a congruent location (i.e., cheek) with attractive vs. non-attractive face, and in the other half they were touched on an incongruent location (i.e., forehead) with attractive vs. non-attractive face.
3. Due to time and practical constraints, there was no implicit measure of enfacement (i.e., self-face recognition test). Instead, the enfacement questionnaire was administered both before (baseline) and after the interpersonal stimulation (post; Figure 6), unlike Experiment 1 where it was only administered post-stimulation. Specifically, participants were presented for 5 s with still images of the attractive and the non-attractive face to obtain a measure of enfacement prior to any interpersonal stimulation. This was repeated twice for each face as a baseline for the two stimulation types (congruent and incongruent).
4. Experiment 1 did not involve any measure of participants’ self-attractiveness, yet this may have influenced the degree of perceived similarity between themselves and the attractive versus the non-attractive face. Therefore, here at the end of the experimental task, participants were asked to complete a short demographic questionnaire, as well as the physical attractiveness item (item number 4) from the Body Image States Scale (BISS; Cash, Fleming, Alindogan, Steadman, & Whitehead, 2002).

#### Data analysis

All statistical analyses were performed using the Statistical Package for the Social Sciences (SPSS) version 23 (IBM, Chicago, IL, USA). Given that repeated measures (Level 1) were nested within individuals (Level 2), multilevel modelling was implemented in exactly the same way as Experiment 1.

### Results

#### Explicit self-report enfacement

##### Overall enfacement

**Figure 7.**
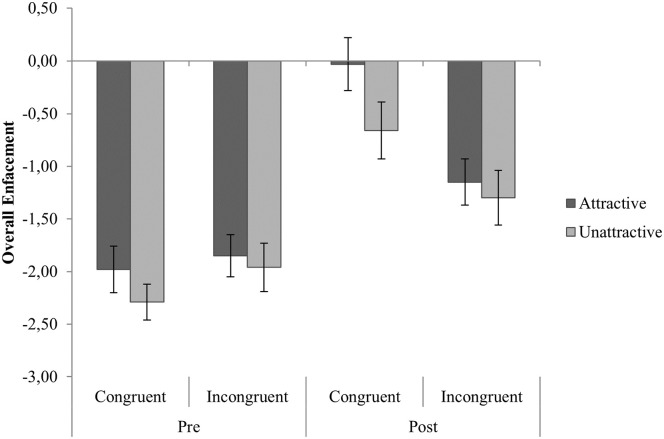
Means for overall enfacement in Experiment 2. Higher scores indicate greater enfacement. Error bars denote standard errors.

The first LMM showed a significant effect of attractiveness on “pre” scores (*b* = 0.311 *SE* = 0.129, *p* = .021), with attractive face (*M* = −1.92, *SD* = 1.26) leading to higher levels of enfacement, as compared with non-attractive face (*M* = −2.13, *SD* = 1.16) as shown in Figure 7.

The results of the second LMM with “post” score as the outcome variable are presented in the table below:

As reported on Table 2, there was a significant main effect of congruency, with congruent stimulation (*M* = −0.346, *SD* = 1.55) leading to higher levels of overall enfacement as compared with incongruent stimulation (*M* = −1.22, *SD* = 1.42). The main effect of attractiveness and the interaction between attractiveness and congruency were non-significant.

**Table 2.** Multilevel modelling results for outcome variable “post” scores.

Effect	*b*	*SE*	*p*-value	95% confidence interval	
				Lower bound	Upper bound
Attractiveness	0.153089	0.185730	.416	−0.224444	0.530622
Congruency	**0.640733**	**0.205812**	**.004**	**0.222641**	**1.058825**
Congruency × Attractiveness	0.482143	0.295539	.112	−0.119504	1.083789
Pre	**0.675441**	**0.083201**	**.000**	**0.510442**	**0.840440**
Self-attractiveness	−0.023515	0.066805	.727	−0.159620	0.112591

*SE*: standard error. Significant main effects and interactions are highlighted in bold.

##### Sub-component analysis: identification

**Figure 8.**
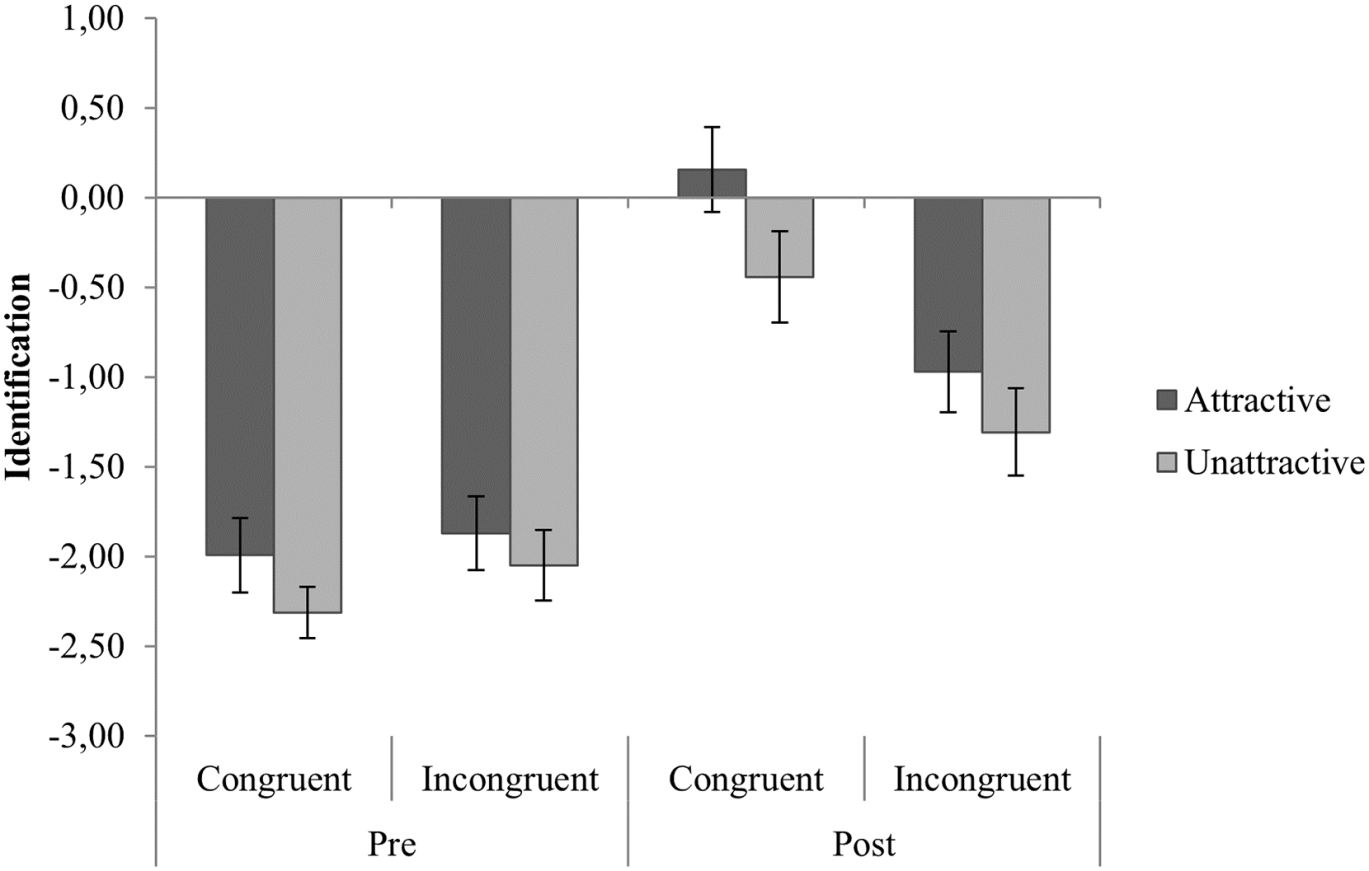
Means for identification in Experiment 2. Higher scores indicate greater identification. Error bars denote standard errors.

The first LMM showed a significant effect of attractiveness on “pre” scores (*b* = 0.250 *SE* = 0.119, *p* = .038), with attractive face (*M* = −1.93, *SD* = 1.21) leading to higher levels of identification, as compared with non-attractive face (*M* = −2.18, *SD* = 1.02) as shown in Figure 8.

The results of the second LMM with “post” score as the outcome variable are presented in the table below:

As reported on Table 3, there was a significant main effect of congruency with congruent stimulation (*M* = −0.143, *SD* = 1.49.) leading to higher levels of identification as compared with incongruent stimulation (*M* = −1.14, *SD* = 1.41.). The main effect of attractiveness and the interaction between attractiveness and congruency were non-significant.

**Table 3.** Multilevel modelling results for outcome variable “post” scores.

Effect	*b*	*SE*	*p*-value	95% confidence interval	
				Lower bound	Upper bound
Congruency	**0.861212**	**0.223246**	**.000**	**0.407413**	**1.315011**
Attractiveness	0.332641	0.195536	.098	−0.065073	0.730354
Congruency × Attractiveness	0.285863	0.310368	.364	−0.345910	0.917637
Pre	**0.602448**	**0.094414**	**.000**	**0.415088**	**0.789809**
Self-Attractiveness	−0.034759	0.070257	.624	−0.177863	0.108346

*SE*: standard error. Significant main effects and interactions are highlighted in bold.

##### Sub-component analysis: similarity

**Figure 9.**
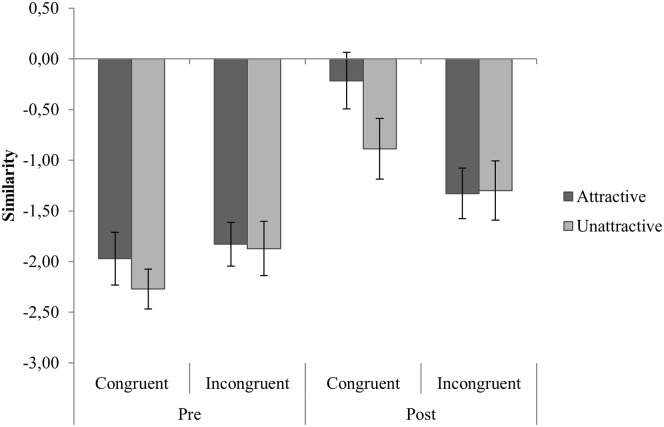
Means for similarity in Experiment 2. Higher scores indicate greater similarity. Error bars denote standard errors.

The first LMM found no main effect of attractiveness on “pre” scores (*b* = 0.171 *SE* = 0.162, *p* = .293, see Figure 9).

The results of the second LMM with “post” score as the outcome variable are presented in the table below:

As reported on Table 4, there was a significant interaction between congruency and attractiveness. Bonferroni-corrected post hoc tests (α = .025) revealed that there was no difference between attractive vs. non-attractive face for perceived similarity neither when the stimulation was congruent, *t*(34) = 2.21, *p* = .034, *d* = 0.392, nor incongruent, *t*(34) = −0.101, *p* = .920, *d* = 0.019.

**Table 4.** Multilevel modelling results for outcome variable “post” scores.

Effect	*b*	*SE*	*p*-value	95% confidence interval	
				Lower bound	Upper bound
Congruency	0.414286	0.213760	.061	−0.019772	0.848343
Attractiveness	−0.028842	0.245289	.907	−0.527052	0.469367
Congruency × Attractiveness	**0.700271**	**0.334618**	**.044**	**0.019141**	**1.381401**
Pre	**0.632506**	**0.082976**	**.000**	**0.467971**	**0.797042**
Self-Attractiveness	−0.018887	0.073598	.799	−0.169009	0.131235

*SE*: standard error. Significant main effects and interactions are highlighted in bold.

In both experiments, we found that the interaction between “attractiveness” and “synchrony” (Experiment 1), as well as “attractiveness” and “congruency” (Experiment 2) was non-significant for identification and significant for similarity (although Bonferroni-corrected post hoc tests in Experiment 2 did not confirm a significant difference between attractive vs. unattractive for congruent stimulation; only a trend towards significance with a medium effect size). To explore the overall effect for identification and similarity, we combined the post-stimulation scores of the two experiments (*N* = 70) and found that the discrepancy between identification and similarity holds across temporal and spatial disparities, that is, significant interaction effect for similarity but not identification (see Supplementary Material D for analyses). The effect size of the interaction for identification was small to medium, whereas the effect size of the interaction for similarity was large.

For the manipulation checks on Trustworthiness and Attractiveness, see Supplementary Material E.

## General discussion

Over two experiments, we investigated for the first time the role of facial attractiveness in the multisensory modulation of face ownership using the enfacement illusion paradigm. First, the important role of multisensory integration in face ownership recognition was confirmed (Paladino et al., 2010; Sforza et al., 2010; Tsakiris, 2008), showing that temporally and spatially congruent visuo-tactile stimulation leads to higher levels of enfacement, measured both at an explicit and implicit level. Most importantly, this study provides the first direct evidence that facial attractiveness has an effect on some facets of face ownership, and this effect seems to be partly independent of multisensory integration processes.

To begin with, the results of both experiments showed that explicit, as well as implicit face ownership was higher for an attractive versus a non-attractive face, even when participants were visually exposed to the face for only 5 s, in the absence of any visuo-tactile stimulation. Moreover, the significant main effects of synchrony/congruency and attractiveness and the absence of an interaction between attractiveness and synchrony/congruency for overall explicit self-report enfacement and identification indicate that attractive faces lead to higher levels of enfacement and identification as compared with non-attractive ones, regardless of any stimulation type, suggesting top–down effects. Nevertheless, the same was not true for perceived similarity (second sub-component). For similarity ratings, and in line with our main hypothesis, an interaction was found between attractiveness and synchrony, as well as between attractiveness and spatial congruency. Combining the data of the two experiments (*N* = 70) showed that the discrepancy between identification and similarity holds across spatial and temporal disparities. This difference in the effect of attractiveness on perceived identification and similarity was also demonstrated by the effect of attractiveness on identification at baseline, purely based on vision, which was not found for similarity. This discrepancy between the two sub-components, which is in line with previous research (Panagiotopoulou et al., 2017), indicates that identification and similarity are possibly mediated by independent mechanisms. In fact, self-identification is considered to be one of the key processes involved in the formation of a mental representation of our physical appearance (Tajadura-Jiménez et al., 2012). This process matches to the identification component of the enfacement questionnaire, referring to a more general matching between felt and seen sensorimotor signals (Tajadura-Jiménez et al., 2012), which, in turn, leads to the formation of a mental representation of one’s physical appearance. However, the sub-component of similarity refers to a more specific experience of physical resemblance with the other person and, hence, it may be considered a distinct process. Our findings, therefore, confirm the importance of studying identification and similarity separately rather than merely obtaining an overall score of enfacement.

It is also important to note that some of the biggest effects were seen in the negative direction (i.e., “not agreeing with the statements”). To begin with, the enfacement illusion is a very subtle phenomenon (Porciello, Bufalari, Minio-Paluello, Di Pace, & Aglioti, 2018). It also important to distinguish between the two experiments. In Experiment 2, this could be due to baseline ratings in the absence of stimulation, which could have biased participants. Future studies could explore the role of different baselines in the final effect. In Experiment 1, we saw positive ratings for attractive face when the stimulation was synchronous and negative ratings for all other conditions. The negative ratings for the two asynchronous conditions were expected and in line with the literature. The negative ratings for the unattractive face in the synchronous condition are of theoretical interest as this could suggest that unattractiveness may actually abolish the effect in synchronous/congruent conditions. Future research is required to explore this further.

Despite the effects of attractiveness being dissociable for different components of face ownership, attractiveness appears to overall enhance face ownership and the possible interpretations regarding the underpinning reasons are multiple. To begin with, one plausible hypothesis of why attractiveness leads to higher levels of identification with another face, over and above any multisensory effects, could be offered from a social psychology perspective. Research has shown that attractiveness leads to imitation, which is a fundamental aspect of the process of identification (Tajadura-Jiménez et al., 2012). For instance, Muller et al. (2013) found that empathy predicts imitation but only for attractive others and not for unattractive. Similarly, Babel, McGuire, & King (2014) found social selectivity in spontaneous phonetic imitation, with the degree to which vowels were imitated being affected by attractiveness ratings. This imitation is thought to stem from the idea that “what is beautiful is good” and the need to affiliate with and take on characteristics and behaviours of people who are beautiful, hence nice (Lakin & Chartrand, 2003; van Leeuwen, van Baaren, Martin, Dijksterhuis, & Bekkering, 2009). As an extension of what social identity theory posits, that is people having a tendency to classify themselves into social categories and favour their in-groups to enhance their self-esteem (Tajfel & Turner, 1979), it is possible that the identification with a more attractive individual serves the adaptive purpose of classifying the self within a category, which is already favoured, therefore, enhancing one’s self-esteem.

Alternatively, another plausible interpretation of why people are inclined to identify more with an attractive face as compared with a non-attractive face after brief visual exposure could be provided by salience-driven attention. Previous research has shown that attractive faces capture greater spatial attention as compared with non-attractive faces, even if the task is unrelated to the judgement of attractiveness (Liu & Chen, 2012; Sui & Liu, 2009). More recently, attractiveness has also been found to temporally modulate visual attention (Nakamura & Kawabata, 2014) and the attention to the attractiveness of a face has been shown to be rapid and automatic (Palermo & Rhodes, 2007; Sui & Liu, 2009). Therefore, the orthogonal effects of attractiveness and multisensory integration on identification could possibly be explained by the fact that processing fluency underlies preference for attractive faces (Reber, Schwarz, & Winkielman, 2004; Trujillo, Jankowitsch, & Langlois, 2014) and, hence, this may be driving visual effects by causing less “prediction” errors in the visual domain. By contrast, perceived similarity with an attractive face may be dependent on temporal and spatial congruency in multisensory integration in that any visual differences between own and other face need to be attenuated for the visuo-tactile input to dominate over proprioceptive information. Of course, these are all exploratory hypotheses so further research is required to disentangle the different mechanisms driving the effects of attractiveness.

Recently, Filippetti, Kirsch, Crucianelli, & Fotopoulou (2018) investigated the role of affective, top–down aspects of sensory congruency between visual and tactile modalities in the sense of body ownership using the rubber-hand illusion (RHI). They found that incongruency between felt and vicariously perceived sensory events led to lower levels of explicit self-report embodiment, irrespective of any valence effect. To test the effect of such an incongruency in the second experiment of this research, a measure of participants’ own perceived attractiveness was obtained to explore the role of perceived attractiveness congruency in enfacement with an attractive vs. non-attractive face. Ratings of self-attractiveness were found to have no effect, suggesting that the top–down aspect of attractiveness congruency does not influence explicit self-report enfacement of an attractive vs. non-attractive face. In other words, participants identified more with the attractive face regardless of how attractive they perceived themselves to be.

Instead, actual physical resemblance could mediate this effect, but one limitation of this study is that the actual physical similarity between participants and the attractive and non-attractive faces was not controlled for. However, this factor is unlikely to have an influence as the findings for the similarity sub-component suggest multisensory effects over and above initial baseline effects. Given the evidence that average faces are rated as more attractive (Valentine, Darling, & Donnelly, 2004), we controlled for distinctiveness, defined as deviation from an average face. Nevertheless, we did not control the physical characteristics of attractive and non-attractive faces; therefore, future research could explore which are the attractive physical characteristics that lead to higher levels of enfacement. Finally, participants were Caucasian female students from a Western university so future studies could explore whether the effects are replicated in a more diverse sample and extend to males too, for whom dominance could potentially have a more pronounced effect as compared with attractiveness, given previous research suggesting that testosterone increases perceived dominance but not attractiveness of male faces (Swaddle & Reierson, 2002). Despite the limitations of this study, we sought to maximise the power, not only by conducting a priori power analyses to determine the sample size, but also by using both an explicit and an implicit measure of enfacement (Experiment 1). In addition, although we used limited faces—one attractive and one non-attractive per experiment—a survey was conducted prior to the experiment with a separate sample of women to identify faces that were significantly different in terms of attractiveness but were matched for all other dimensions (trustworthiness, dominance, and distinctiveness). In the second experiment, a new set of attractive and non-attractive faces was used to ensure that any effects were not specific to the faces of the first experiment.

To conclude, previous research has revealed a self-enhancement bias for attractiveness, with people perceiving themselves as more attractive than others consider them to be. The findings of this study suggest that this self-enhancement bias could be mediated by others’ facial attractiveness, through blurring of self–other boundaries. More specifically, the results showed that others’ attractiveness can influence the relatively automatic and perceptual process of face ownership. Increased ratings of attractiveness of a new, unfamiliar face lead to identification of our psychological self with another’s physical self, and more specifically their face. Although higher levels of identification with another attractive face seem to be driven by vision, reflecting a more general, top–down effect of attractiveness, the effect of attractiveness on similarity appears to be dependent on multisensory integration so future research could try to disentangle the exact mechanism behind these effects. In a society where we are deluged with images of attractive people through the media, our findings suggest that others’ attractiveness may actually lead to identification with the more rather than the less attractive others. Yet, the question remains as to how such “positive distortions” of the self can affect one’s psychological well-being. This research may provide a psychophysical starting point for studying the impact of others’ attractiveness in self-perception, which can be particularly important for individuals with malleable, embodied self–other boundaries and body image disturbances.

## Supplemental Material

sj-docx-1-qjp-10.1177_17470218211050318 – Supplemental material for Identifying with the beautiful: Facial attractiveness effects on unisensory and multisensory self–other distinction Click here for additional data file. Supplemental material, sj-docx-1-qjp-10.1177_17470218211050318 for Identifying with the beautiful: Facial attractiveness effects on unisensory and multisensory self–other distinction by Elena Panagiotopoulou, Laura Crucianelli, Alessandra Lemma and Aikaterini Fotopoulou in Quarterly Journal of Experimental Psychology
