# Supplementary material for: Identifying with the beautiful: Facial attractiveness effects on unisensory and multisensory self–other distinction
Source: Q J Exp Psychol (Hove). 2021 Oct 13;75(7):1314–29. doi: 10.1177/17470218211050318 (PMC9131399; doi:10.1177/17470218211050318)
Supplement: sj-docx-1-qjp-10.1177_17470218211050318 – Supplemental material for Identifying with the beautiful: Facial attractiveness effects on unisensory and multisensory self–other distinction [file sj-docx-1-qjp-10.1177_17470218211050318.docx]

Supplementary Material for

**Identifying with the Beautiful:**

**Facial attractiveness effects on unisensory and multisensory self-other distinction**

Elena Panagiotopoulou, Laura Crucianelli, Alessandra Lemma & Aikaterini Fotopoulou

**Supplementary Material A:** Facial Attractiveness Survey in Exp. 1

The 65 participants of this survey (mean age = 28.68, SD = 11.64; all females) were presented with 25 Caucasian female faces and were asked to rate on a scale from 0 (not at all) to 100 (extremely) how “attractive”, “trustworthy”, “dominant” and “distinctive” each face was. Those 25 faces were from an unselected, consecutive sample of women who had previously taken part in another experiment, were unknown to current participants and had given their written permission for their faces to be used in this new experiment. These attributes were chosen as they have been found to be distinct properties that influence attractiveness ratings. Specifically, trustworthiness and dominance were selected given that they are thought to be primary dimensions of face evaluation influencing social judgments (Oosterhof & Todorov, 2008). Distinctiveness, defined as deviation from an average face, was also controlled for, given previous research showing that there is a complex relationship with attractiveness, with unattractive faces being distinctive, and attractive faces being rated at all levels of distinctiveness (Wickham & Morris, 2003). For the current sample of 25 faces, the mean rating for attractiveness was 37.66 (SD = 11.46), trustworthiness 42.44 (SD = 7.93), dominance 38.36 (SD = 5.70) and distinctiveness 41.76 (SD = 4.61). The two faces selected to represent attractive and non-attractive faces differed significantly in perceived attractiveness (p < .001) but not in the other three attributes (p > .05).

|  | *Attractiveness* | *Trustworthiness* | *Dominance* | *Distinctiveness* |
| --- | --- | --- | --- | --- |
| **Attractive** | 63.29 (22.29) | 46.26 (22.87) | 40.22 (21.84) | 43.22 (21.10 ) |
| **Non-attractive** | 24.91 (19.98) | 38.61 (22.97) | 36.21 (19.83) | 39.14 (22.64) |

**Table A.1:** Means (and standard deviations) from 65 raters for selected faces in Experiment 1

**Supplementary Material B:** Manipulation checks for trustworthiness and attractiveness in Exp. 1

*Trustworthiness*

A 2x2 ANOVA revealed a significant main effect of “attractiveness” [F (1,34) = 18.23, p < .001, η^ 2^ = .349] with attractive face producing higher levels of trustworthiness as compared to non-attractive face. A trend was found for “synchrony” [F (1,34) = 4.01, p = .053, η^ 2^ = .105] with synchronous stroking producing higher levels of trustworthiness as compared to asynchronous stroking. The interaction between “synchrony” and “attractiveness” was not significant [F (1,34) = 1.200, p = .324, η^ 2^ = .029] (Figure B.1).

**Figure B.1**: Means for trustworthiness in Exp. 1. Higher scores indicate greater trustworthiness. Error bars denote standard errors

*Attractiveness*

A 2x2 ANOVA revealed a significant main effect of “attractiveness” [F (1,34) = 26.34, p < .001, η^ 2^ = .459] with attractive face being rated as more attractive than the non-attractive face. A trend was found for “synchrony” [F (1,34) = 3.21, p = .083, η^ 2^ = .094] with synchronous stroking producing higher levels of attractiveness as compared to asynchronous stroking. The interaction between “synchrony” and “attractiveness” was not significant [F (1,34) = .897, p = .351, η^ 2^ = .028)] (Figure B.2).

**Figure B.2**: Means for attractiveness in Exp. 1. Higher scores indicate greater attractiveness. Error bars denote standard errors.

**Supplementary Material C:** Facial Attractiveness Survey in Exp. 2

The 25 participants of this survey (mean age = 25.42, SD = 9.43; all females) were presented with 11 Caucasian female faces and were asked to rate on a scale from 0 (not at all) to 100 (extremely) how “attractive”, “trustworthy”, “dominant” and “distinctive” each face was. Those 11 faces were from an unselected, consecutive sample of women who had previously taken part in Experiment 1, were unknown to current participants and had given their written permission for their faces to be used in this new experiment. For the current sample of 11 faces, the mean rating for attractiveness was 43.99 (SD = 13.81), trustworthiness 49.74 (SD = 5.66), dominance 50.38 (SD = 8.25) and distinctiveness 56.67 (SD = 9.60). The two faces selected to represent attractive and non-attractive faces differed significantly in perceived attractiveness (p < .001) but not in the other three attributes (p > .05) (see Table C.1 for details). Moreover, they were matched for eye and hair colour (blond hair, blue eyes).

|  | *Attractiveness* | *Trustworthiness* | *Dominance* | *Distinctiveness* |
| --- | --- | --- | --- | --- |
| **Attractive** | 60.00 (15.63) | 50.00 (15.49) | 49.17 (13.57) | 46.67 (12.11) |
| **Non-attractive** | 30.00 (6.32) | 45.00 (10.00) | 47.50 (15.41) | 50.00 (16.73) |

**Table C.1:** Means (and standard deviations) for attribute scores for selected faces for Experiment

**Supplementary Material D:** Overall effect for identification/similarity across spatial and temporal disparities.

To investigate the overall effect for identification and similarity across spatial and temporal disparities, we combined the post-stimulation data of the two experiments (N=70) and repeated-measures analysis of variance (ANOVA) was performed on the subcomponent scores with Temporal/Spatial Congruency (congruent vs. incongruent) and Attractiveness (attractive vs. non-attractive) as within-subject factors. Bonferroni corrected post-hoc analyses were conducted when appropriate.

For identification, we found a significant main effect of temporal/spatial congruency [F (1,69) = 81.58, p < .001, η^2^ = .542], with congruent stimulation (M = -.016, SE = .147) leading to higher levels of enfacement as compared to incongruent stimulation ((M = -1.08, SE = .137). We also found a significant main effect of attractiveness [F (1,69) = 10.96, p = .001, η^2^ = .137], with attractive face (M = -.346, SE = .137) leading to higher levels of enfacement as compared to non-attractive face (M = -.748, SE = .149). The interaction between temporal/spatial congruency and attractiveness was non-significant [F (1,69) = 1.91, p = .171, η^2^ = .027].

For similarity, we found a significant main effect of temporal/spatial congruency [F (1,69) = 41.01, p < .001, η^2^ = .373], with congruent stimulation ((M = -.225, SE = .166) leading to higher levels of enfacement as compared to incongruent stimulation (M = -1.039, SE = .169). A significant main effect of attractiveness [F (1,69) = 6.89, p = .011, η^2^ = .091] was also found, with attractive face (M = -.421, SE = .167) leading to higher levels of enfacement as compared to non-attractive face (M = -.843, SE = .183). The interaction between temporal/spatial congruency and attractiveness was also significant [F (1,69) = 9.41, p = .003, η^2^ = .120]. Bonferroni-corrected post hoc tests (alpha = 0.025) revealed that perceived similarity was higher for attractive vs. unattractive face when the stimulation was temporally and spatially congruent [t (69) = 3.61, p = .001, d = .457] but not incongruent [t (69) = .524, p = .602, d = .062].

**Supplementary Material E:** Manipulation checks for trustworthiness and attractiveness in Exp. 2

*Trustworthiness*

**Figure E.1***:* Means for similarity in Exp. 2. Higher scores indicate greater trustworthiness. Error bars denote standard errors.

The first LMM found a significant main effect of attractiveness on ‘pre’ score (*b* = 1.37 *SE* = .205, *p* < .001), with attractive face (M = .857, SD = 1.44) leading to higher levels of trustworthiness, as compared to unattractive face (M = -.514, SD = 1.54, see Figure E.1).

The results of the second LMM with ‘post’ score as the outcome variable are presented in the table below:

|  | | | | | | |
| --- | --- | --- | --- | --- | --- | --- |
| Effect | b | SE | p-value | 95% Confidence Interval | |  |
|  |  |  |  | Lower Bound | Upper Bound |  |
| *Congruency* | -.228571 | .315744 | .474 | -.870570 | .413428 |  |
| ***Attractiveness*** | **1.114286** | **.257329** | **.000** | **.591545** | **1.637027** |  |
| *Congruency x Attractiveness* | .342857 | .289842 | .246 | -.249029 | .934743 |  |
| ***Pre*** | **.530370** | **.062000** | **.000** | **.407283** | **.653457** |  |
| *Self-Attractiveness* | -.038794 | .058993 | .515 | -.158777 | .081190 |  |

**Table E.1:** Multilevel modelling results for outcome variable ‘post’ scores. Significant main effects and interactions are highlighted in bold.

As reported on Table E.1, there was a significant main effect of attractiveness with attractive face (M = .1.34, SD = 1.36.) leading to higher levels of trustworthiness as compared to non-attractive face (M = .057, SD = 1.63.). The main effect of congruency and the interaction between attractiveness and congruency were non-significant.

*Attractiveness*

**Figure E.2***:* Means for similarity in Exp. 2. Higher scores indicate greater attractiveness. Error bars denote standard errors.

The first LMM found a significant main effect of attractiveness on ‘pre’ score (*b* = 2.36 *SE* = .168, *p* < .001), with attractive face (M = .743, SD = 1.30) leading to higher levels of attractiveness, as compared to unattractive face (M = -1.61, SD = 1.27, see Figure E.2).

The results of the second LMM with ‘post’ scores as the outcome variable are presented in the table below

|  | | | | | | |
| --- | --- | --- | --- | --- | --- | --- |
| Effect | b | SE | p-value | 95% Confidence Interval | |  |
|  |  |  |  | Lower Bound | Upper Bound |  |
| *Congruency* | .057143 | .168203 | .736 | -.282873 | .397159 |  |
| ***Attractiveness*** | **2.285714** | **.178182** | **.000** | **1.926651** | **2.644778** |  |
| *Congruency x Attractiveness* | .085714 | .208995 | .685 | -.340415 | .511843 |  |
| ***Pre*** | **.661801** | **.055862** | **.000** | **.551026** | **.772575** |  |
| *Self-Attractiveness* | -.026214 | .043821 | .556 | -.117219 | .064792 |  |

**Table E.2:** Multilevel modelling results for outcome variable ‘post’ scores. Significant main effects and interactions are highlighted in bold.

As reported on Table E.2, there was a significant main effect of attractiveness with attractive face (M = .986, SD = 1.23.) leading to higher levels of attractiveness as compared to non-attractive face (M = -1.343, SD = 1.33.). The main effect of congruency and the interaction between attractiveness and congruency were non-significant.
